# Lennard–Jones Parameter Fitting for Gold/Water Interaction Based on Structural Analysis: A QM, MM, and QM/MM Study

**DOI:** 10.3390/nano16030160

**Published:** 2026-01-24

**Authors:** Pere Bancells i Blazquez, Federico Nicolás Pedron, Anthoni Alcaraz Torres, Elizane Efigenia de Moraes, Ivan Cole, Ernane de Freitas Martins

**Affiliations:** 1 Catalan Institute of Nanoscience and Nanotechnology-ICN2 (CSIC and BIST), 08193 Bellaterra, Barcelona, Spain; pere.bancellsi@autonoma.cat (P.B.i.B.); federico.pedron@bsc.es (F.N.P.); anthoni.alcaraz@icn2.cat (A.A.T.); 2 Facultat de Ciències, Universitat Autònoma de Barcelona, 08193 Bellaterra, Barcelona, Spain; 3 Instituto de Física, Universidade Federal da Bahia, Salvador 40210-340, Bahia, Brazil; elizanemoraes@ufba.br; 4 School of Engineering, Australian National University (ANU), Canberra, ACT 2601, Australia; ivan.cole@anu.edu.au

**Keywords:** Lennard–Jones, interfaces, molecular modeling, DFT, QM/MM, classical MD, fitting

## Abstract

The interaction between water and metallic interfaces is crucial in many fields, and accurate modeling requires good parametrizations using reference data. In classical molecular dynamics (MD), an important part of this interaction is described using the Lennard–Jones (LJ) potential. However, previously reported LJ parameters are not always optimal for capturing the metal/water interactions observed in ab initio descriptions such as density functional theory (DFT). Therefore, well-tailored LJ parameters are necessary to improve the description of water structuring metals in classical MD. The usual route for obtaining LJ parameters involves energetic analysis, where the energies of various structures are obtained via DFT calculations and then matched with the energies obtained using the LJ potentials by varying the sigma/epsilon parameters. Here, we show a different approach to fit LJ parameters for metal/water interactions, based on structural analysis. We report several classical MD simulations for gold/water, varying the sigma/epsilon parameters, comparing the resulting water structuring with that obtained using DFT. Additionally, we test these parameters in quantum mechanics/molecular mechanics (QM/MM) MD simulations, where electrostatic interactions are enabled. Our results demonstrate that the proposed approach can improve the LJ parameters reported in the literature and potentially develop parameters for more complex systems where the water structure above metallic surfaces plays a significant role. Finally, within this proposed approach, the water density profile obtained in hybrid QM/MM calculations, where water is treated as MM at a substantially reduced cost, closely matches the description it would have if treated as QM.

## 1. Introduction

Describing water/metallic interfaces is relevant for many applications, including materials science, physical chemistry, electronic devices, and detection technologies. Understanding these interactions at the molecular level is essential for predicting and describing the behavior of complex systems, which in turn drives advances in applications such as chemical sensors, electrochemical devices, and catalytic processes. In recent decades, computational simulations have gained significant importance due to the increasing availability of computer hardware and software resources. These simulations validate existing theoretical frameworks and also enable the exploration of complex systems that are challenging or impossible to study experimentally with atomic resolution. As a result, molecular modeling has become indispensable in fields such as materials science and physical chemistry, where a deep understanding of the atomic and electronic properties and behaviors of substances is critical for both fundamental research and practical applications [1,2,3,4,5,6,7,8,9,10,11,12].

In atomistic molecular modeling, two main approaches exist, differing in the way the system components are modeled: quantum mechanics (QM) or ab initio, which considers electron and nuclei behavior derived from quantum mechanics principles [13], and molecular mechanics (MM), which considers atoms as the smallest individual unit and uses classical physics to derive interactions between the atoms [14]. The first one should provide a more exact and rigorous solution, but its computational cost is high and is often unaffordable. MM is usually used instead to get faster results. This approach, despite its known limitations, yields satisfactory results and, with the necessary precautions, can effectively reproduce the QM behavior. The low computational cost of molecular dynamics (MD) based on MM, known as classical MD, allows it to be applied to larger, more complex systems over longer timescales [15] than those reached by MD based on QM, known as ab initio MD. Hybrid methods such as QM/MM [16] have been developed to bridge the gap between predictability and computational efficiency. These methods combine the rigor of QM with the simplicity and low cost of MM, enabling the study of systems where quantum effects are localized but classical approximations suffice for the rest of the system [17].

In this context, fitting parameters for atomistic potentials, particularly Lennard–Jones (LJ) parameters, is crucial for accurately describing metal/water interactions in classical MD simulations. Typically, classical atomistic potentials are parameterized to reproduce macroscopic properties extracted from experimental data. However, this approach may be insufficient because a potential parameterized to reproduce a specific macroscopic property of a bulk system will not necessarily provide good results for a mixture or interface. Quantum effects and the collective behavior of water are relevant near interfaces, and these effects can be obtained via ab initio MD based on density functional theory (DFT) simulations.

For example, Moraes et al. [18] developed a force field for benzene using DFT to capture the most stable configurations of benzene dimers. This model reproduced the dynamics, diffusion, phase transitions, glassy phase, and supercritical region behaviors of benzene [18,19]. More recently, machine learning models using the Deep-MD technique, where the database is based on ab initio calculations, have been successful in reproducing the bulk phase properties of water [20,21]. However, due to the complexity of force field (FF) parameterization, these models have not been implemented in confined systems or interfaces. In this context, new force field parameterization strategies are needed to efficiently capture QM information from interfaces at a low computational cost, for example, when relying on methods such as QM/MM. This hybrid approach can describe the metal quantum mechanically while including the water molecules, which are typically the most computationally demanding part due to the significant number of atoms, via parametrized classical FF. If such parametrization utilizes water structural information from QM, then QM/MM simulations can provide a more accurate description of metal/water interactions.

Comprehensive studies on the development and parametrization of force fields for metal atoms are scarce, although most databases include parameters for them [22]. However, these parameters are subject to specific restrictions or assumptions that may render them unsuitable for all systems. These constants are often defined using a system energy minimization approach, which is appropriate for bulk-like systems. However, for purposes such as corrosion studies, the spatial arrangement of water molecules plays a crucial role [11], necessitating structurally appropriate parameters. Furthermore, the spatial arrangement of water is critical to the structure of the electrified double layer at a charged surface in solution, affecting both the inner and outer Helmholtz layers.

Some approaches based on structural properties have been developed for other types of complexes, such as cysteine and selenocysteine systems [23]. R1 Q2: Structural fitting over energy-based fitting can improve matching ab initio and classical MD water layering. However, the high cost of ab initio might limit this approach, albeit modern DFT codes and supercomputer facilities make it possible. Still, there is a lack of research on how to apply these methods specifically to metal/water interfaces. In the case of gold, research exists on the optimization of LJ parameters for interactions with water using energy-based approaches [24]. However, despite their importance in device applications, no studies have focused on the structural characteristics of water distribution near gold surfaces. Addressing these gaps through improved parametrization, including the use of QM/MM methods to further improve the simulations, could significantly enhance the accuracy and applicability of simulations involving metal/water interfaces.

In this work, we conducted a systematic study to optimize LJ parameters for gold/water interactions using structural analysis. We compared the classical MD water structure for different sets of LJ parameters with that obtained via ab initio MD. We further check the quality of the optimized LJ parameters by performing QM/MM hybrid MD runs. This approach aims to enhance the description of water structuring above metallic surfaces, expanding the scope of parameter optimization beyond traditional energy-based methods.

## 2. Methodology

### 2.1. Lennard–Jones Interactions

In QM/MM, the energy contributions can typically be split between those of the QM region, the MM region, and the QM-MM cross-term. In the case of this method [25,26,27] implemented in the SIESTA [28,29] code, the cross-term energy is given by

(1) $$E_{QM/MM}=\sum_{i=1}^{N_{MM}}q_{i}\int \frac{\rho (\vec{r})}{|\vec{r}-{\vec{\tau }}_{i}|}dr\;+\sum_{i=1}^{N_{MM}}\sum_{j=1}^{N_{QM}}\frac{q_{i}Z_{j}}{{\vec{R}}_{j}-{\vec{\tau }}_{i}}\;+\;E_{LJ}\;,$$

where *j* stands for the QM and *i* stands for the MM atoms, $\rho (\vec{r})$ is the electronic density of the QM atoms, $q_{i}$ is the partial charge of the MM atoms, $Z_{j}$ is the atomic charge of the QM atoms, $N_{QM}$ is the total number of QM atoms, $N_{MM}$ is the total number of MM atoms, and $E_{LJ}$ is a Lennard–Jones (LJ) potential computed as

(2) $$E_{LJ}=4\varepsilon_{ij}\sum_{i=1}^{N}\sum_{j=1}^{N}\left[{\left(\frac{\sigma_{ij}}{r_{ij}}\right)}^{12}-\;{\left(\frac{\sigma_{ij}}{r_{ij}}\right)}^{6}\right]\;,$$

where $\varepsilon_{ij}$ and $\sigma_{ij}$ are parameters fitted for each pair of i,j interactions, which are, in the case of QM/MM, QM and MM atoms. For purely classical MD simulations, Equation (2) is also frequently used (like in this work) to treat van der Waals interactions, where i,j would refer exclusively to MM atoms. In Equation (2), $r_{ij}$ is the distance between each pair of i,j and $\varepsilon_{ij}$ is the energy at the equilibrium distance $r_{min}$. This $r_{min}$ is related to σ as

(3) $$r_{min,ij}=2^{1/6}\sigma_{ij}\;,$$

and the mixing rules [22] for $r_{min,ij}$ and $\varepsilon_{ij}$ are

(4) $$r_{min,ij}=\frac{r_{min,ii}+r_{min,jj}}{2}\;\mathrm{and}\;\varepsilon_{ij}=\sqrt{\varepsilon_{ii}\;\varepsilon_{jj}}\;,$$

where ii and jj refer to individual ε and $r_{min}$ for each atom. Here, we consider the Modified Transferable Interaction Potential with 3 Points (mTIP3P) [30,31] model for water, which includes LJ interactions for H and O atoms. In this way, for classical MD, there will be intermolecular LJ interactions according to the above equations for H-H, O-O, and O-H pairs. For gold, there will be LJ interactions for Au-Au, Au-H, and Au-O pairs. In the convention adopted by the SIESTA code, we input the individual LJ atomic parameters ($\varepsilon_{ii}$ and $\frac{r_{min,ii}}{2}$), and one can easily recover another code’s convention by applying Equation (3). In the paper, we will refer to the vdW LJ parameters as σ and ε to simplify the notation.

In the particular case of this work, where our system is composed of a gold slab in contact with liquid water, the $\varepsilon_{ii}$ and $\frac{r_{min,ii}}{2}$ parameters entered in the input file correspond to the Au-Au, H-H, and O-O pairs. All remaining pair parameters are computed according to Equation (4). Taking this into account, we have performed several classical MD simulations varying Au-Au σ and ϵ parameters. The resulting water distribution profiles were compared against the one obtained from ab initio MD to obtain the one deviating the least, thus fitting LJ parameters to ab initio water distributions above gold slabs. We then compared the behavior of QM/MM MD simulations when using both our optimized LJ parameters and those found in the literature [24]. In this paper, we will refer to the MD simulations as QM, MM, or QM/MM MD.

### 2.2. Comparing Water Distributions

The MM water distribution profiles obtained for the varying LJ parameters were compared to the QM profiles using the (1) root mean square deviation between slopes (${\mathrm{RMSD}}_{\mathrm{slope}}$) and the (2) Jaccard similarity coefficient (J(A,B)). In the ${\mathrm{RMSD}}_{\mathrm{slope}}$ method, we can focus on the most relevant part of the distribution profiles, which is the distance from the gold slab [23,32]. Therefore, we can establish a limited computation of the RMSD, and the equation used is given by

(5) $${\mathrm{RMSD}}_{\mathrm{slope}}=\sqrt{\frac{1}{N_{A}}\sum_{i=a}^{N_{A}}{\left(\rho_{\mathrm{MM}}\left(z_{i}\right)-\rho_{\mathrm{QM}}\left(z_{i}\right)\right)}^{2}+\frac{1}{N_{B}}\sum_{i=b}^{N_{B}}{\left(\rho_{\mathrm{MM}}\left(z_{i}\right)-\rho_{\mathrm{QM}}\left(z_{i}\right)\right)}^{2}}\;,$$

where $\rho_{\mathrm{QM}}$ and $\rho_{\mathrm{MM}}$ are the water densities obtained from QM and MM simulations, respectively, and $N_{A}$ and $N_{B}$ are the numbers of data points considered in the relevant regions of the distribution.

We have also utilized the J(A,B). This statistical parameter enables us to verify whether two curves match correctly, as used in various fields such as meteorology or ecology [33]. It has many different definitions, but the essential basis lies in comparing the intersection of the sets and their union. In this context, it consists of the ratio between the area that the two distributions share and the total area occupied when the functions overlap, given by

(6) $$J\left(A,B\right)=\frac{\left|A\cap B\right|}{\left|A\cup B\right|}=\frac{A_{\mathrm{intersection}}}{A_{\mathrm{union}}}=\frac{A_{\mathrm{intersection}}}{A+B-A_{\mathrm{intersection}}}\;,$$

where *A* and *B* represent the areas under the curves of the water distributions obtained from QM and MM simulations, respectively.

While the ${\mathrm{RMSD}}_{\mathrm{slope}}$ assures that sampling issues are screened (peak heights can be different and will not affect results), J(A,B) guarantees a similar area of the selected region as well as a similar shape (peak heights and widths can be different and will not affect results). In the case of ${\mathrm{RMSD}}_{\mathrm{slope}}$, the smaller the parameter obtained, the closer the MM and QM curves are, whereas for J(A,B), the closer the value is to 1, the closer the MM and QM curves are.

### 2.3. Computational Details

The Au/water ab initio MD used as a reference for fitting the classical MD LJ parameters was performed using DFT with DRSLL functional [34], DZP basis set, Meshcutoff of 200 Ry, and Troulier–Martins pseudopotentials [35] to treat the core electrons in the psf format. We chose the DRSLL functional because it includes van der Waals interactions, and is reported in the literature to treat liquid water adequately [36]. For the classical MD, water molecules were described using the mTIP3P [30,31] model. Regarding the LJ parameters, we tested the range shown in Table 1 and the values reported in the literature [24].

In the case of QM/MM simulations, all gold atoms were quantum mechanically treated (QM region). In contrast, the water molecules, representing the most extensive part of the system, are treated via classical force fields (MM region). These descriptions coincide with the levels of theory used in both fully QM and fully MM simulations. In the case of gold atoms, we used the LJ parameters from the literature and those that yielded the best results from our fitting.

**Figure 1:**
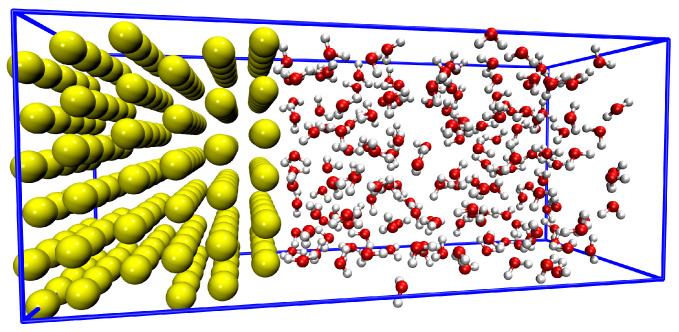
Side view in perspective of the XZ plane for the system investigated. The system contains 180 Au atoms and 236 water molecules, totaling 888 atoms. The golden, white, and red spheres represent Au, H, and O atoms.

All QM, MM, and QM/MM MD simulations followed an annealing protocol from 0 K to 300 K using a 20 fs relaxation time and 1 fs time step, with a total sampling time of 30 ps. However, all Au atoms were frozen during the simulations to facilitate an accurate comparison of all the trajectories. The system used for all simulations, containing 180 Au atoms and 236 water molecules, totaling 888 atoms, is shown in Figure 1. The box x,y,z dimensions are 15.714 Å × 15.120 Å × 61.759 Å, and the Au slab used is cleaved in the 111 crystallographic plane. The initial structure and velocities were the same for all MD simulations. Both QM/MM and MM simulations employed a cutoff distance of 8 Å for non-bonded interactions and a real-space list of neighbors. All simulations were performed using the SIESTA [28,29] code. R1 Q6: The solvate tool of the Gromacs [37] software was used to add the water molecules to the system, providing the initial solvation for further MD using SIESTA. All distribution analyses were performed using self-made C++ scripts.

## 3. Results

### 3.1. Reference QM and MM Data

**Figure 2:**
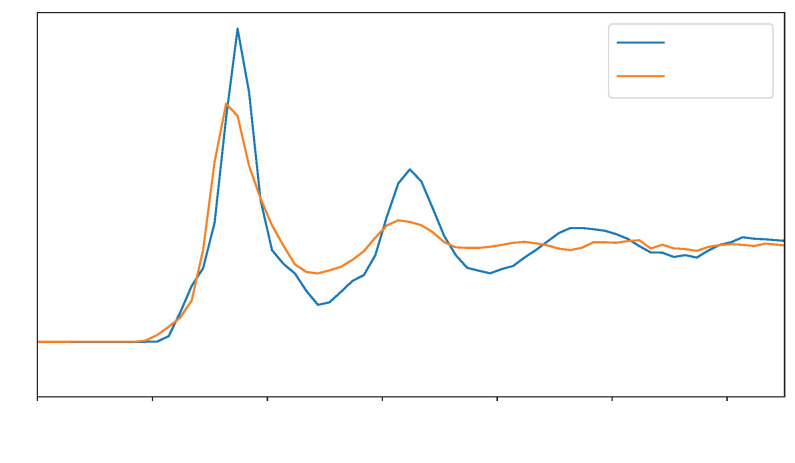
Water distribution profile comparison between our quantum mechanics (QM) reference and the molecular mechanics (MM) run using Lennard–Jones parameters from [24]. Only the left-side region is shown for clarity. The full profile and the separate H and O curves are available in Appendix A Figure A1.

We initially ran a QM MD calculation to be used as our reference to fit the LJ parameters. The structure of water above metals has already been revealed in the literature [24,38]. More specifically, in the case of Au, water is known to exhibit at least two prominent peaks near the interface, followed by bulk-like behavior with no apparent structuring. This structuring for water above gold is observed for both QM and MM simulations, and this is precisely what we observe in Figure 2. In all of the water distribution plots shown in this paper, we show only the surface on the left side for the sake of clarity. The full-range graphics are presented in Appendix A and are found to be completely symmetric, as expected.

**Figure A1:**
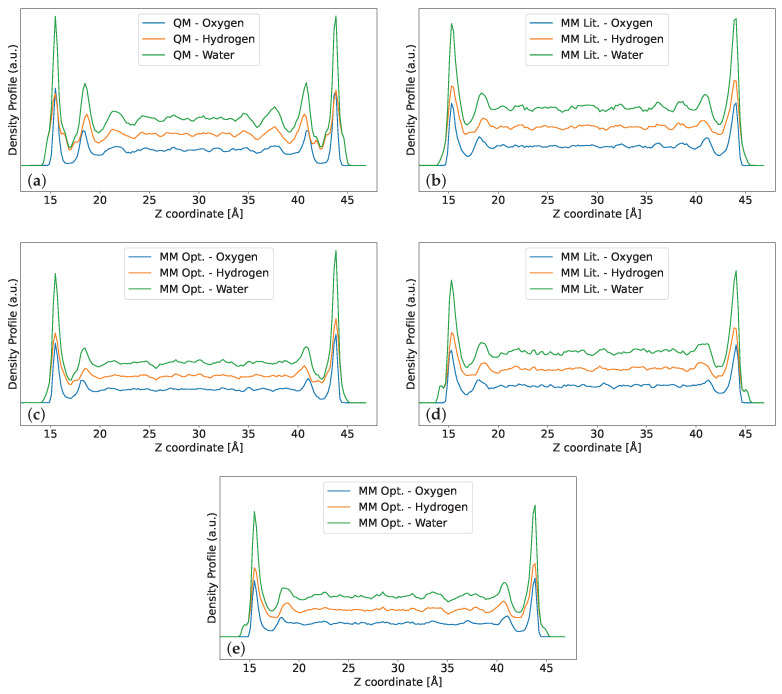
Full range for water distribution profiles for all simulations shown in the main text for (**a**) reference quantum mechanics (QM), (**b**) reference molecular mechanics (MM), (**c**) optimized MM, (**d**) reference quantum mechanics/molecular mechanics (QM/MM), and (**e**) optimized QM/MM.

As can be seen by comparing the curves obtained from QM and MM MD using the LJ parameters from [24] in Figure 2, some differences are observed. First, the QM curve presents a small shoulder near the interface due to the interactions between the H and Au atoms (see Appendix A Figure A1 for separate H and O water distribution profiles). Another difference can be observed in the MM MD curve at the position of the first solvation peak, indicating that the water molecules are closer to the interface than those in the QM one. The first difference is primarily due to electrostatic interactions, which are absent in the fully classical MD runs since the partial charges on the Au atoms are set to zero. The second is mainly controlled by the σ and ε LJ parameters. As such, obtaining LJ parameters that decrease the differences observed in Figure 2 might provide water distributions closer to our reference QM result.

**Figure A2:**
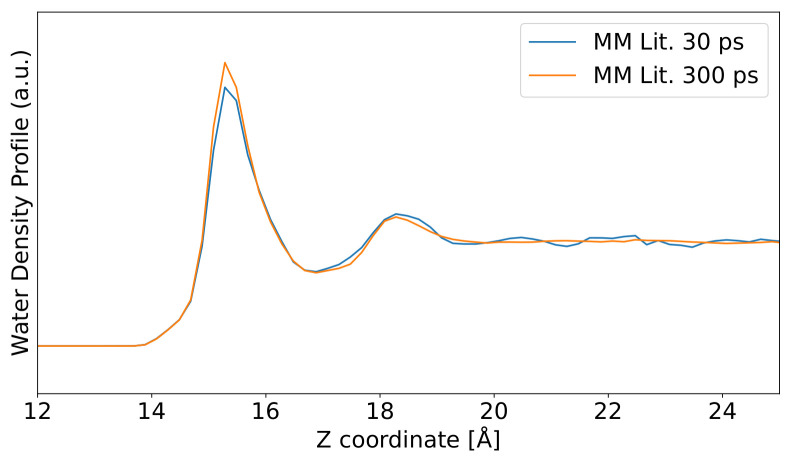
Water distribution profiles comparison for molecular mechanics (MM) curves. The blue line shows the average water distribution for a 30 ps long run, like all the other runs in the manuscript, and the orange one shows the distribution for a 10 times bigger (300 ps) run. The LJ parameters used for both MM runs are taken from [24].

Furthermore, the blue curve in Figure 2 shows a third peak for QM water. The first peak of water will be the one most affected by the interaction with Au. The remaining peaks will mostly depend on water/water interactions. In this case, fitting LJ parameters for Au will have little to no effect on those peaks. R1 Q2: In this way, our method is limited to reproducing only the first solvation peak, and further improvement can be done employing different water models, which is out of the scope of this paper. Finally, we also observe that the height of the first MM water peak does not agree with that of the QM distribution, indicating that the MM run underestimates the number of water molecules forming the first solvation layer. R1 Q4: A longer classical MD run demonstrating that 30 ps are enough to have an equilibrated water distribution is shown in Appendix A Figure A2, also showing that the slopes of the first solvation peak are virtually unchanged.

### 3.2. Lennard–Jones Parameters Fitting

We then proceeded to run several MM MD simulations for varying LJ parameters. We chose a range of 1.3–2.2 in steps of 0.1 Å for $\frac{r_{min}}{2}$ (called σ here to simplify notation; see Equation (3)) and 0.8–2.2 in steps of 0.1 kcal mol^−1^ for ϵ, as shown in Table 1. For each pair of σ and ε, we evaluated how the obtained MM MD water distribution curve deviated from our QM reference. We have chosen the region between 13.0 Å and 17.0 Å as the definition for the first solvation layer of water (see Figure 2). Then, the two comparison techniques used here (${\mathrm{RMSD}}_{\mathrm{slope}}$ and J(A,B)) are used in this area.

**Figure 3:**
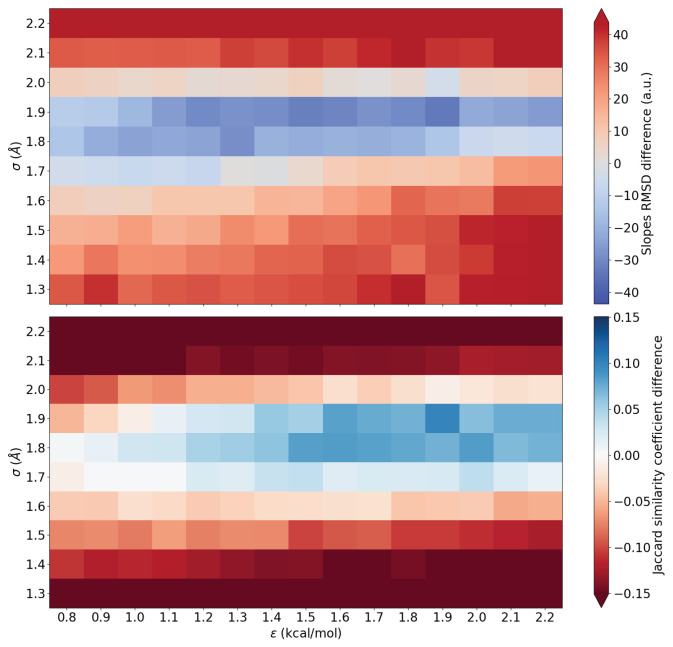
${\mathrm{RMSD}}_{\mathrm{slope}}$ (above) and J(A,B) (below) results for varying σ and ε. For clarity, the zero value (white/gray color) corresponds to the reference taken from [24].

To provide a more graphical explanation, we chose to visualize the data using color maps. The plots shown in Figure 3 use zero (white/gray color) as the RMSD difference between the MM MD using the values from the literature [24] and the reference QM run. For ${\mathrm{RMSD}}_{\mathrm{slope}}$, blue means that there is a smaller RMSD (i.e., better) between the chosen MD and the reference QM distribution when compared to the LJ parameters found in literature. Meanwhile, for J(A,B), the closer the number is to one, the better the result, and we chose blue for that.

R1 Q3: Therefore, Figure 3 shows the results for the two comparison methods, where we considered that the first slope (ascendant) comprises the region between 0.4 and 0.1 Å before the QM water distribution first peak maximum and the second one (descendant) includes the region between 0.1 and 0.4 Å after the QM water distribution first peak maximum. This window should be chosen according to each system in such a way that it comprises the two slopes. Yet, evaluating the slopes avoids longer simulations usually required to sample peak heights adequately, while their slopes do not change significantly [23]. Thus, the darker the blue region, the better the LJ parameter pair.

As can be seen in Figure 3, there is a continuous range of parameter pairs that provide significantly better results than the rest (lower values of RMSD in the above panel of Figure 3). This region encompasses values from nearly all the ε values tested, but it is better in the σ interval that goes from 1.7 Å to 2.0 Å. The same behavior is observed for the J(A,B) results (below panel of Figure 3). We can observe the same pattern in the same region as that observed for the ${\mathrm{RMSD}}_{\mathrm{slope}}$ method, but it appears to be slightly shifted towards larger values of ε. Therefore, there is a group of parameters that better fit the QM water distribution.

**Figure 4:**
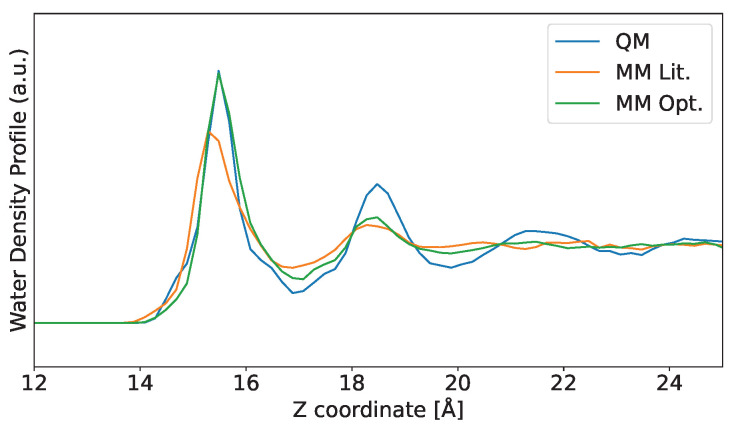
Water distribution profiles comparison for the optimized Lennard–Jones (LJ) parameters, showing the reference quantum mechanics (QM) and molecular mechanics (MM) curves again for comparison purposes. The LJ parameters used for the MM run, as cited in the literature, are taken from [24]. Only the left-side region is shown for clarity. The full profile and the separate H and O curves are available in Appendix A Figure A1.

In this way, we can conclude that both ${\mathrm{RMSD}}_{\mathrm{slope}}$ and J(A,B) methods are equally suitable for obtaining the LJ parameters using our reference QM water distribution. Therefore, the outcome of the two methods is that the parameter pair with the best results is the one with ε=1.9 kcal/mol and σ=1.9 Å. In Figure 4, the water distribution obtained using the new optimized LJ parameters is shown along with the previous curves already presented (QM and MM MD using LJ parameters from the literature [24]).

As one can see in Figure 4, both height and position for the first solvation peak are in better agreement with the QM curve (compare green and blue lines) than that using the parameters found in literature (orange curve). Yet, we notice little difference for the second solvation peak, as expected, because only the first solvation peak will have a strong interaction with the Au surface. As mentioned before, the subsequent solvation peaks will be affected mainly by the water/water interactions, which are determined by the FF employed for water in the case of MM simulations.

### 3.3. QM/MM Approach

**Figure 5:**
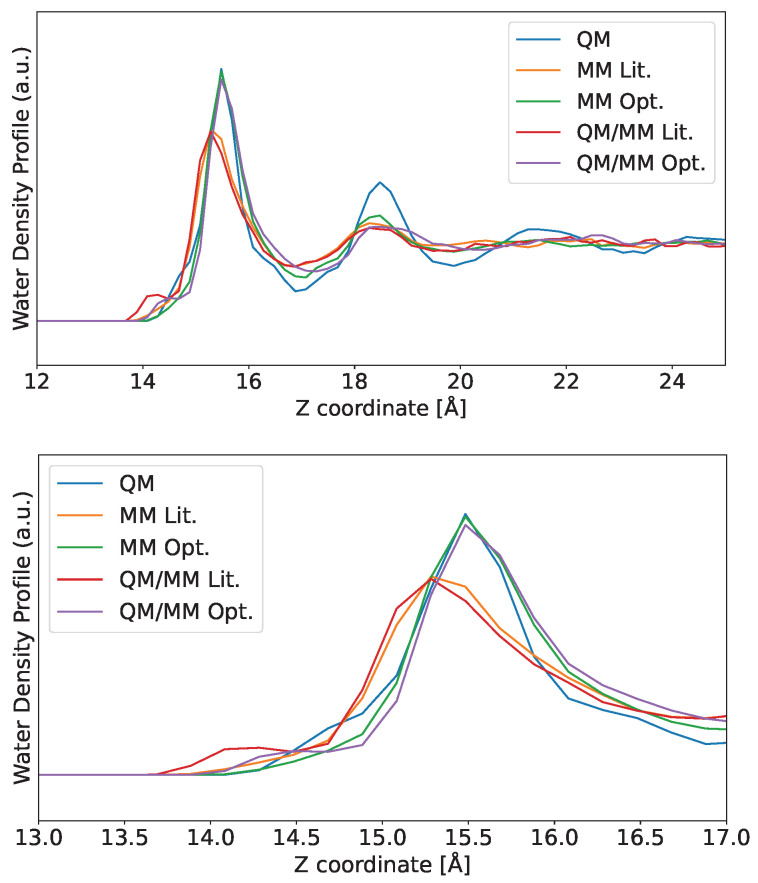
Water distribution profiles for all the models used: reference quantum mechanics (QM), literature and optimized molecular mechanics (MM), and literature and optimized quantum mechanics/molecular mechanics (QM/MM). The reference QM and MM curves are shown again for comparison purposes. The Lennard–Jones parameters used for the MM and QM/MM runs from the literature are taken from [24]. The panel below shows a zoom of the first solvation peak region to improve the view. Only the left-side region is shown for clarity. The full profile and the separate H and O curves are available in Appendix A Figure A1.

Having MM water distribution matching QM distribution is particularly important for hybrid QM/MM simulations; in particular, the distribution profile of MM water above QM Au slabs will be determined mainly by the choice of the LJ potential (see Equation (1)). We performed QM/MM MD using both the literature [24] and our optimized LJ parameters, and the results are shown in Figure 5. We also present the curves for classical MD simulations for comparison purposes.

The water profiles shown in Figure 5 demonstrate that the QM/MM distribution closely follows the MM one. This result shows the importance of fitting LJ parameters, as the curves for the optimized values are in much better agreement with the QM one. The importance of choosing the appropriate LJ parameter is highlighted when considering that, in the QM/MM method (see Equation (1)), there is an electrostatic interaction, and yet the solvent distribution is heavily influenced by the choice of LJ parameters. Finally, enabling electrostatic interactions for the QM/MM curves recovered the H-attraction by the Au atoms observed in the reference QM simulation, albeit slightly overestimated. Even then, using the LJ parameters optimized in this work, both MM and QM/MM water distribution profiles achieve results that closely follow the full QM distribution.

R1 Q5 and R2 Q1: Finally, it is worth discussing transferability and limitations of the proposed fitting approach. The parameters provided in this work were fitted for Au(111) and mTIP3P water, and are not necessarily transferable to other facets, mobile Au slabs, or different water models without further validation. Also, freezing Au atoms enabled consistent QM, MM, and QM/MM comparisons but can also limit transferability. However, these are not limitations unique to our optimized parameters. Indeed, some of their limitations are shared with all pairwise LJ models, including parameter sets from the literature that were fitted under similarly restricted assumptions. Our parameters, like other fitted data reported in the literature, should not be assumed to be broadly transferable. Finally, as we mentioned before, the lack of Coulombic interactions of the classical MD calculations used by us is recovered by our QM/MM simulations.

## 4. Conclusions

In this work, we have found new σ and ϵ Lennard–Jones (LJ) parameters for Au that better fit with ab initio molecular dynamics (MD) results for water structuring above the surface. To do so, we have performed several classical MD runs for varying LJ parameters and compared the resulting water profile distribution with an ab initio reference. We have employed two different comparison methods: (1) least-squares fitting of the first peak slopes (${\mathrm{RMSD}}_{\mathrm{slope}}$) and (2) Jaccard similarity index (J(A,B)) calculation for the same zone on the peak of the first solvation layer. Both provide similar results when using optimal parameters.

We then performed QM/MM MD simulations using the LJ parameters fitted in this work and those found in the literature, with our parameters resulting in classical and QM/MM MD that closely follow the ab initio reference. The water distribution obtained using the new LJ parameters showed a clear improvement compared to those found in the literature. As a side note, the QM/MM runs show that enabling electrostatic interactions among Au and water recovers the H-attraction shoulder in the water distribution, albeit slightly overestimating it. R1 Q2: Nonetheless, our method is limited to reproducing only the first solvation peak, and transferability to other Au facets or systems with mobile atoms should be considered before re-using such parameters and the proposed method.

We conclude that the improvement found in the description of water distribution above Au surfaces was worth the effort of re-fitting the LJ parameters. Moreover, we believe that the methodology shown here represents a step forward in obtaining parameters to simulate metallic/water interfaces, enabling higher-quality simulations.

## Figures and Tables

**Table 1:** LJ parameters used in this work according to the SIESTA code convention. $\frac{r_{min}}{2}$ is presented as the input in the SIESTA code, and it relates to σ according to Equation (3).

Atom Pair		$\frac{r_{\min}}{2}$ (Å)	ε (kcal mol^−1^)
H-H	[31]	0.2245	0.0460
O-O	[31]	1.7683	0.1521
Au-Au	[24]	1.6450	1.0516
Au-Au	[This work]	1.3–2.2, in steps of 0.1	0.8–2.2, in steps of 0.1

## Data Availability

The raw data supporting the conclusions of this article will be made available by the authors upon request.

## Abbreviations

The following abbreviations are used in this manuscript:

QM	Quantum mechanics
MM	Molecular mechanics
QM/MM	Quantum mechanics/molecular mechanics
MD	Molecular dynamics
DFT	Density functional theory
LJ	Lennard–Jones
RMSD	Root mean square deviation
vdW	Van der Waals
mTIP3P	Modified transferable interaction potential with 3 points
SIESTA	Spanish initiative for electronic simulations with thousands of atoms
